# Genetic Screening of a Nonsyndromic Amelogenesis Imperfecta Patient Cohort Using a Custom smMIP Reagent for Selective Enrichment of Target Loci

**DOI:** 10.1155/humu/8942542

**Published:** 2025-07-22

**Authors:** Ummey Hany, Christopher M. Watson, Lu Liu, Georgios Nikolopoulos, Claire E. L. Smith, James A. Poulter, Agne Antanaviciute, Alice Rigby, Richard Balmer, Catriona J. Brown, Anesha Patel, María Gabriela Acosta de Camargo, Helen D. Rodd, Michelle Moffat, Gina Murillo, Amal Mudawi, Hussain Jafri, Alan J. Mighell, Chris F. Inglehearn

**Affiliations:** ^1^Leeds Institute of Medical Research, University of Leeds, St. James's University Hospital, Leeds, UK; ^2^North East and Yorkshire Genomic Laboratory Hub, Central Lab, St. James's University Hospital, Leeds, UK; ^3^School of Dentistry, Clarendon Way, University of Leeds, Worsley Building, Leeds, UK; ^4^Institute for Fundamental Biomedical Research, B.S.R.C. ‘Alexander Fleming', Attica, Greece; ^5^MRC Human Immunology Unit, University of Oxford, Oxford, UK; ^6^Birmingham Dental Hospital, Mill Pool Way, Birmingham, UK; ^7^LCRN West Midlands Core Team, NIHR Clinical Research Network, Birmingham, UK; ^8^Department of Paediatric Dentistry, School of Dentistry, Universidad de Carabobo, Valencia, Venezuela; ^9^Academic Unit of Oral Health Dentistry and Society, School of Clinical Dentistry, University of Sheffield, Sheffield, UK; ^10^Paediatric Dentistry, The Newcastle upon Tyne Hospitals NHS Foundation Trust, Newcastle upon Tyne, UK; ^11^School of Dentistry, Universidad de Costa Rica, Ciudad Universitaria Rodrigo Facio, San Pedro, Montes De Oca, Costa Rica; ^12^Elrazi University, Khartoum, Sudan; ^13^University of Kalisz, Kalisz, Poland

**Keywords:** amelogenesis, enamel development, tooth disease

## Abstract

Amelogenesis is the process of tooth enamel formation, and genetic variants disrupting it cause the Mendelian inherited disorder amelogenesis imperfecta (AI). AI patients have weak, discoloured or brittle enamel, caused by reduced enamel quantity or mineralisation. AI can occur in isolation or, less commonly, as part of a syndrome. Pathogenic variants in at least 38 genes have been shown to cause AI. Current genetic screening studies typically use exome sequencing, but this is expensive and involves complex data analysis workflows. Target enrichment using smMIPs (single molecule molecular inversion probes) provides a flexible alternative, allowing the creation of a disease-specific reagent for low cost, robust, high-throughput screening. Here, we describe the development of an smMIP reagent targeting 19 genes implicated in isolated AI and assess its use in screening a cohort of 181 UK probands with nonsyndromic AI. While this was intended only as a prescreen to prioritise exome sequencing more efficiently, it nevertheless led to molecular diagnoses for 63 probands (35%). Cost per sample screened was approximately £40. Variants in three genes, *COL17A1*, *FAM83H* (both dominant) and *MMP20* (recessive), accounted for approximately half of solved cases. There is scope to further improve the smMIP reagent by adding additional probes targeting regions of low coverage or additional genes, including those involved in syndromic AI, as well as accommodating new information about the genetic basis of AI. The smMIP reagent provides a robust, flexible, high-throughput, low-cost approach to AI screening, and it is available as a resource to the international AI research community.

## 1. Introduction

Amelogenesis describes the process of tooth enamel formation. Ameloblasts, which are derived from the oral epithelium, form a monolayer around the developing enamel. The highly coordinated sequence of expression of key genes by ameloblasts is essential for the formation and mineralisation of enamel during tooth development. Molecular disruption of amelogenesis is the mechanism underlying amelogenesis imperfecta (AI), a Mendelian inherited disorder affecting the enamel appearance, quantity, quality and function of all teeth of both dentitions. AI can result in weak, discoloured enamel that easily breaks down, or a reduced enamel volume, with no enamel formed in the most extreme instances. It can occur in isolation or as a component of a series of syndromic conditions. It can be difficult to distinguish clinically between syndromic and isolated AI, reflecting the fact that additional clinical features can be subtle or of variable severity or timing in their clinical presentation. It can also be challenging to distinguish AI from other developmental defects of enamel. Within these limitations, the reported prevalence of isolated AI ranges from one in 233 in Turkey [1] to one in 700 in Sweden [2], one in 1000 in Argentina [3], one in 8000 in Israel [4] and one in 14,000 in the United States [5]. Since it was first discovered that mutations in the X-linked gene *AMELX* (amelogenin, X) cause isolated AI [6], a further 20 autosomal genes have been reported to be associated with isolated AI [7, 8]. Of these 21 genes, pathogenic variants in one cause X-linked disease, in 10 cause autosomal recessive (AR) disease, in eight cause autosomal dominant (AD) disease and in two can cause both dominant and recessive forms of isolated AI.

Determining which gene and variant(s) cause AI in patients gives a clear prognosis, informs management (which may include genetic counselling for patients and relatives) and increases our understanding of underlying biological mechanisms, supporting future research. For more than a decade, next generation sequencing (also known as massively parallel or clonal sequencing) has revolutionised the availability, speed and accuracy of diagnostic screening for pathogenic variants causing inherited conditions [7, 9]. Although whole exome sequencing (WES) is less expensive than whole genome sequencing (WGS), it is still relatively costly compared to customised ‘targeted' approaches. Furthermore, WES and WGS analysis pipelines are computationally demanding; data storage has governance, operational and cost implications; and both assays have the potential to generate coincidental findings.

Target enrichment using single molecule molecular inversion probes (smMIPs) [10] presents an attractive alternative to WES/WGS, allowing selective screening of specific genes or loci in large patient cohorts. Originally developed for targeted genotyping of SNPs (single nucleotide polymorphisms) in patients with immunoglobin nephropathy or Berger's disease [11], its application now spans a wide range of fields, from clinical genetics to evolutionary biology. It has also been used successfully for diagnosing a wide range of inherited conditions, including patients with *ABCA4*-associated Stargardt disease, macular dystrophy and male infertility [12–15].

smMIPs are oligonucleotide probes consisting of a common DNA backbone flanked by target-specific sequences known as the ligation and extension arms. Probes hybridise to the complementary target genomic sequence of the arms. The arms act as primers, allowing a DNA polymerase to close the gap between them. The product is then circularized by DNA ligase. The circular DNA is subsequently linearized and amplified by PCR using universal primers complementary to the probe backbone. Thousands of probes can be mixed in a single reaction to amplify multiple target regions from a single DNA sample. Samples can be multiplexed through the addition of short unique index sequences to the primer, allowing the identification of sample-specific reads [16]. The technique has been further improved by incorporating unique molecular identifiers (UMIs) with the potential to capture single molecular events [10]. One recent study used smMIPs to screen exons spanning over 450 kb of genomic sequence, at loci distributed throughout the genome, in 300 patients in a single sequencing run [17]. This approach provides a lower cost per sample than other comparable targeted analysis workflows [10].

Here, we describe the development of a custom smMIP reagent for screening genomic DNA from patients with isolated AI and demonstrate its utility in identifying molecular diagnoses in previously undiagnosed cases. The smMIP reagent was designed to capture the coding sequences and immediate splice donor and acceptor sites of 19 genes associated with isolated nonsyndromic AI. The assay was validated using control samples that had previously been analysed by WES, then used to screen 181 unsolved cases from an isolated-AI cohort.

## 2. Materials and Methods

### 2.1. Participant Recruitment

Patients with a clinical diagnosis of isolated AI were recruited from multiple UK collaborating centres, with informed written consent and ethical approval (REC 13/YH/0028) in accordance with the principles of the Declaration of Helsinki. Inclusion criteria were a confirmed AI diagnosis after examination by a dental specialist. Exclusion criteria included unclear diagnosis, secondary enamel defects due to systemic illness (e.g., coeliac disease) or environmental causes (e.g., fluorosis) or evidence of a syndromic condition. Genomic DNA was isolated from saliva using Oragene DNA sample collection kits (DNA Genotek) following the manufacturer's protocol.

### 2.2. Gene Selection and smMIP Design

Probes were designed using MIPGEN (https://github.com/shendurelab/MIPGEN) [18]. Each probe was designed to have an approximately 82 nucleotide backbone (including the extension and ligation arms located at each end) and a 110 nucleotide region-specific target sequence. Extension and ligation arms together were 45 nucleotides long and were complementary to sequences adjacent to the region-specific target sequence. The common linker sequence joining the two arms contained universal PCR primer sites, followed by an eight nucleotide stretch of random bases. The latter provided a degenerate molecular index with 4^8^ possible unique combinations for each amplicon [19]. Probe selection was performed by visualising all candidate probes on the Integrative Genome Viewer (IGV) [20]. For each target region, a single probe was selected, targeting either the plus or minus strand of the DNA with a minimum 10 bp overlap with adjacent probes wherever possible. Probes with high logistic scores, as calculated by MIPGEN, were selected where possible. Five hundred seventeen of the 609 probes used had logistic scores ≥ 0.5. Probes with scores of less than 0.5 were used in challenging genomic regions including the *FAM83H* and *ACP4* loci [10]. The details of the loci and smMIP sequences are provided in Table S1.

### 2.3. Probe Preparation

Each smMIP was synthesised at a 100 nanomole scale (in 96-well plate format) without modifications (Integrated DNA Technologies). Probes were then pooled to an equimolar concentration to create a ‘megapool' for hybridisation to genomic DNA. A 25 *μ*L aliquot of the megapool was 5⁣′-phosphorylated by adding 1 *μ*L (10 units) of T4 polynucleotide kinase (New England Biolabs, NEB) and 3 *μ*L of 10× T4 DNA ligase reaction buffer with 10 mM ATP. Total volume was made up to 30 *μ*L with 1 *μ*L nuclease-free water. The reaction was incubated at 37°C for 45 min then 65°C for 20 min [16].

### 2.4. Library Preparation and Sequencing

For one affected patient from each of the 181 families investigated, 100 ng genomic DNA in 10 *μ*L nuclease-free water was subjected to targeted hybridisation and ligation using the phosphorylated probe megapool. The probe megapool was diluted to obtain a ratio of 800 MIP copies per single DNA copy in the final capture reaction. A 15 *μ*L hybridisation capture mastermix was prepared, on ice, for each sample. This comprised 2.5 *μ*L of Ampligase 10× reaction buffer (Epicenter), 0.32 *μ*L of dNTP mix (0.025 mM) (NEB), 0.32 *μ*L Hemo KlenTaq (10 U/*μ*L) (NEB), 0.2 *μ*L of Ampligase (5 U/*μ*L) (Epicenter), 3.29 *μ*L of the smMIPs phosphorylated megapool (diluted by a factor 10^5^) and a nuclease-free water to make a total volume of 15 *μ*L. Fifteen microlitres of hybridisation mastermix was then added to 10 *μ*L of genomic DNA (100 ng). The reaction was incubated on a benchtop thermocycler at 95°C for 3 min then 22 h at 65°C. The reaction was then exonuclease treated using a mastermix that contained 0.5 *μ*L of Exonuclease I (NEB), 0.5 *μ*L of Exonuclease III (NEB), 0.2 *μ*L Ampligase 10× reaction buffer (Epicenter) and 0.8 *μ*L of nuclease-free water. The reaction was incubated at 37°C for 45 min then 95°C for 2 min. Following exonuclease treatment, 10 *μ*L of the sample was added to 15 *μ*L of a postcapture PCR mastermix, which was prepared by combining 12 *μ*L of Q5 Hot Start HiFi 2× mastermix (NEB), 1.25 *μ*L of 10 *μ*M forward primer, 1.25 *μ*L of 10 *μ*M barcoded reverse primer and 0.5 *μ*L of nuclease-free water. Thermocycling conditions comprised an initial step of 98°C for 30 s, followed by 23 cycles of 98°C for 10 s, 60°C for 30 s and 72°C for 30 s, then a final extension step at 72°C for 2 min. The reactions were then purified using a 0.8× Axygen AxyPrep MAG PCR clean-up kit, and the fragment distribution of the resulting library was visualised using the DNA 1000 HS assay on a Tapestation (Agilent Technologies, Wokingham, United Kingdom). Each library was individually quantified using a Qubit 2.0 Fluorometer (Invitrogen) and HS DNA reagents. Libraries were then pooled in equimolar concentration for sequencing [16]. Depending on the number of samples processed in an individual batch, sequencing was carried out using either a MiSeq (Illumina Inc.) or a NextSeq 500 (Illumina Inc.) to generate paired-end 150 bp reads. Manufacturers' instructions were followed throughout.

### 2.5. smMIP Probe Rebalancing

The smMIP panel was optimised over five iterative test runs. The mean read depth was calculated for each probe from the previous test run. This value was used to adjust the volume of each underperforming or overperforming probe in the subsequent run, either increasing or decreasing its concentration in the probe megapool. A small number of probes failed; alternative probes were designed to replace these in subsequent runs.

### 2.6. Data Processing Pipeline

An in-house bioinformatics pipeline was developed to process the raw sequence data. For each patient, MIPVAR v.0.1.0 (https://sourceforge.net/projects/mipvar/) was used to process consecutive read-pairs by removing the UMI, then aligning the sequence read to the human reference genome (hg19). The ligation and extension arms were trimmed to eliminate erroneous variant calls caused by hybridisation bias. Read pairs containing identical UMIs that aligned to the same genomic position were marked as PCR duplicates using Picard v.1.119 (https://broadinstitute.github.io/picard/). Nonreference bases were identified and recorded in variant call format (VCF) using the Genome Analysis Toolkit's HaplotypeCaller v.3.7-0 [21]. Each per-patient VCF file was annotated with functionally relevant biological information and observed population frequency data using Annovar [22]. ExomeDepth v1.1.12 [23] was used to perform CNV analysis. We ran an in-house batch analysis script to process samples that were sequenced concurrently on the same machine following the same bioinformatics workflow.

### 2.7. Variant Interpretation

The pathogenicity status of identified variants was classified according to American College of Medical Genetics and Genomics (ACMG) criteria using the online platform Franklin by Genoox (https://franklin.genoox.com/clinical-db/home) [24]. Allele frequencies were obtained from the Genome Aggregation Database v.2.1.1 (https://gnomad.broadinstitute.org/) [25]. In silico splicing predictions were generated using SpliceAI (https://spliceailookup.broadinstitute.org) [26].

### 2.8. Variant Verification and Segregation Analysis

Primers were designed using AutoPrimer3 (https://github.com/david-a-parry/autoprimer3) and synthesised by IDT (Leuven, Belgium). Twenty-five nanograms of genomic DNA was amplified using Q5 High-Fidelity 2× Master Mix (NEB) according to the manufacturer's instructions. 2.5 *μ*L of PCR products were purified using 1 *μ*L ExoSAP-IT (Applied Biosystems). The sequencing reaction mix was prepared by adding 1 *μ*L of ExoSAP-IT treated DNA to a mastermix containing 6 *μ*L of nuclease-free water, 0.5 *μ*L of BigDye Terminator v.3.1 (Applied Biosystems), 1.5 *μ*L of BigDye Terminator v.3.1 Sequencing Buffer (Applied Biosystems) and 1 *μ*L of primer (1.6 *μ*M). Following an initial denaturation step at 96°C for 1 min, the samples underwent 25 cycles of 96°C for 10 s, 50°C for 5 s and 60°C for 4 min. All temperatures were ramped at 1°C/s. Sequencing templates were precipitated using 125 mM EDTA and 100% ethanol, followed by centrifugation at 3900 rpm for 30 min at 4°C. DNA was washed with 70% ethanol and dried at 37°C for 1 min. Precipitates were dissolved in 10 *μ*L Hi-Di Formamide (Applied Biosystems) ready for sequencing. Sequencing was carried out on an ABI3130xl Genetic Analyser (Applied Biosystems) following the manufacturer's instructions. Electropherograms were analysed using SeqScape v.2.5 (Applied Biosystems).

## 3. Results

Nineteen genes associated with isolated AI (Table 1) were selected on the basis that variants in them accounted for the majority of solved AI cases in both the published literature and our own unpublished data at that time. Other genes implicated in nonsyndromic AI are large, while variants in them causing AI were rare, so their inclusion would have substantially diluted the reagent without significantly improving the success rate. These 19 genes were targeted with 609 smMIPs covering the coding exons and immediate splice site sequences. After probe rebalancing, a mean depth of 97% at greater than 20 reads was achieved across the targeted sequences in the 19 genes for the control DNA samples. The optimised reagent was then used to screen genomic DNA from eight validation control samples and 181 probands from unrelated families with isolated AI. The variants identified in each patient were filtered to exclude those with a CADD score < 15 or a minor allele frequency (MAF) > 0.01 for biallelic and > 0.001 for monoallelic variants [25, 27]. The variant list for each case was interpreted to assign classifications of pathogenic, likely pathogenic or variant of unknown significance (VOUS), according to the ACMG criteria. All potentially pathogenic genotypes were resequenced by Sanger sequencing, and their segregation with disease was assessed in all available family members.

### 3.1. Validation Samples

To validate the smMIPs library preparation method and bioinformatics pipeline, eight control DNAs with known pathogenic variants were analysed. These were from three individuals with AI due to homozygous variants (*MMP20* NM_004771.4:c.955A>T p.(Ile319Phe), *KLK4* NM_004917.5:c.632del p.(Leu211Argfs^∗^37) and *RELT* NM_152222.2:c.164C>T p.(Thr55Ile)) and five from individuals with dominant AI due to heterozygous variants (*ENAM* NM_031889.3:c.92T>G p.(Leu31Arg), *LAMB3* NM_000228.3:c.2660G>A p.(Arg887His), *COL17A1* NM_000494.4:c.3595G>C p.(Glu1199Gln), *FAM83H* NM_198488.5:c.1354C>T p.(Gln452^∗^) and *FAM83H* NM_198488.5:c.1192C>T p.(Gln398^∗^)). The smMIP variant calling pipeline correctly identified all these variants in the corresponding samples, showing that the reagent and protocol are effective in screening for a range of variants causing AI.

### 3.2. Patient Screening

Once validated, the reagent was used to screen a cohort of 181 probands with isolated AI. These included 25 families presenting with dominantly inherited AI, 48 with confirmed recessive AI and a further 29 with suspected recessive AI, as well as 79 cases either with no family history or where family history was unknown. No families presented with an unambiguous X-linked family history.

**Figure 1:**
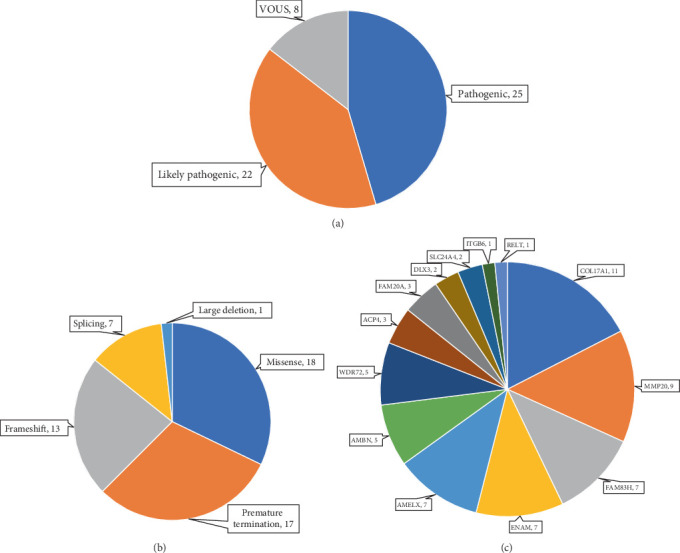
The classification and type of variants detected by smMIP screening of the reported AI cohort. (a) A total of 56 potentially disease-causing variants were detected in 63 families. According to the ACMG classification, 25 of these are classified as pathogenic, 22 are likely pathogenic and eight are variants of uncertain significance (VOUS). (b) The types of mutations detected in smMIPs-AI cohort screening. Of the 56 disease-causing variants detected in 63 families, 18 are missense, 13 are frameshift, 17 are nonsense mutations predicted to lead to a premature termination codon and seven are predicted to alter splice sites and a large deletion. (c) Genetic diagnosis of the AI cohort by smMIP screening. The most commonly identified genes with variants were *COL17A1* (11), *MMP20* (nine), *FAM83H* (seven) and *ENAM* (seven). The numbers of observations in each category are given next to it.

A total of 56 variants were prioritised as candidate disease-associated variants and confirmed by Sanger sequencing in probands and additional family members, where available. These consisted of 18 missense, 17 premature termination, 13 frameshift and seven splice site variants and one large deletion. By ACMG criteria, 25 of these were classified as pathogenic, 22 as likely pathogenic and eight as VOUSs (Tables 2, 3, and 4 and Figure 1a,b). These data resulted in possible or probable molecular diagnoses explaining the condition in 63 probands (35%) by identifying potentially pathogenic genotype combinations in known AI-associated genes.

Of these 63 probands, seven (11%) were found to have X-linked AI (Table 2), 29 (46%) dominant AI (Table 3) and 27 (43%) recessive AI (Table 4). The seven cases solved as X-linked AI had variants in *AMELX*, the only known gene causing AI on the X chromosome. Of the 29 dominant families, *COL17A1* (11 probands) and *FAM83H* (seven probands) gene variants accounted for most cases, with smaller numbers having pathogenic heterozygous variants in *ENAM*, *AMBN* and *DLX3*. No likely dominant disease-causing variants were identified in *LAMB3*, *SP6* or *AMTN* in this cohort. Among probands solved as recessive AI, *MMP20* variants accounted for a relatively large proportion (a third) of cases. This was primarily due to the presence of previously reported common founder variants in the local community [42]. Smaller numbers of variants were also found in *WDR72*, *ACP4*, *FAM20A*, *AMBN*, *SLC24A4*, *RELT*, *ITGB6* and *ENAM*. No variants were identified in three other genes implicated in AR AI, *ODAPH*, *GPR68* and *KLK4*. Variants in *ENAM* and *AMBN* were implicated in both dominant and recessive AI in this cohort. The proportion of families solved by variants in each gene is displayed in Figure 1c.

### 3.3. Possible Digenic Cases

**Figure 2:**
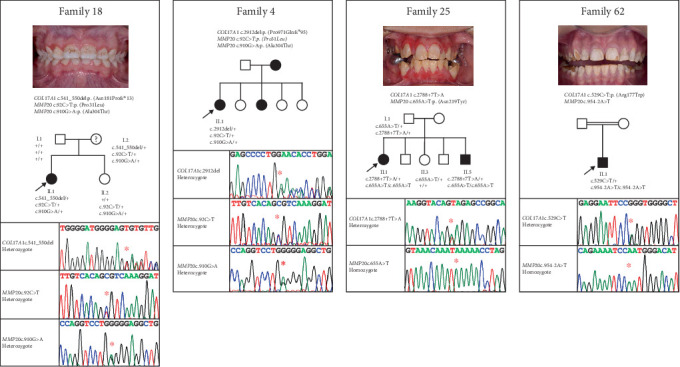
Pedigrees of four families with *COL17A1* and *MMP20* variants and an anterior view of the dentition in the proband where available. Sanger sequencing chromatograms from the proband for each family are displayed beneath each pedigree. A question mark in the pedigree denotes an individual with possible AI who has not been clinically assessed. Probands (indicated with a black arrow) from Families 4 and 18 were diagnosed as having pitted and hypoplastic AI (no image is available from Family 4), consistent with AI due to a dominant heterozygous *COL17A1* variant, and are reported elsewhere [52]. Probands from Families 25 and 62 were diagnosed as having hypomineralised AI, consistent with AI due to recessive *MMP20* variants. +, wild-type allele.

Interestingly, four families presented with potentially pathogenic variants in both *COL17A1* and *MMP20* (Table 5). In each case, families were considered solved based on the presence of a genetic variant or variants within one of these genes correlated to the clinical features, but variant(s) in the other gene were also present and segregated with AI where this information was available. Pedigrees of these families, Sanger sequencing chromatograms from each proband and anterior intraoral images of the teeth where available are displayed in Figure 2.

## 4. Discussion

This study describes the development, validation and assessment of a custom smMIP sequencing reagent targeting the coding exons and splice sites of 19 genes known to harbour pathogenic variants presenting as isolated AI, and its use in screening a single affected participant from each of a cohort of 181 unsolved AI families. Data analysis and storage are simplified with this targeted screening approach, and ethical issues posed by coincidental findings are reduced. The targeted reagent and optimised method used here proved rapid and robust, detecting all validation control variants and solving a third of AI probands screened in a single sequencing run. It was also cost-effective, with a per-sample cost of approximately £40. We anticipate being able to achieve further economies of scale through subsequent rounds of optimisation and greater multiplexing of samples. By comparison, a diagnostic screen for AI available to UK AI patients through the NHS costs over £900 (personal communication), though it should be noted that this includes the cost of genomic interpretation and the issuing of an accredited diagnostic report.

Previous AI cohort studies have suggested that screening all the currently known AI-associated genes solves 50%–60% of cases [48, 49]. The smMIP screen described here identified likely causative variants in 36% of cases and families. However, 89 of the families included in this screen were unsolved after previous screening by Sanger sequencing of genes newly implicated in AI as they were discovered. The success rate in these families was lower (26%) than in new untested cases (43%), suggesting some solvable cases had been excluded prior to smMIP analysis. Furthermore, the smMIP screen was not intended to provide a comprehensive screen, but rather to act as a prescreen for AI cases for known genes, allowing targeting of more comprehensive but costly and labour-intensive WES or WGS to cases unsolved in the initial screen. It is likely that the diagnostic success achieved with the approach described could be improved. The use of smMIPs gives flexibility, allowing design improvements as knowledge advances. Small gaps were noted in the coverage of several genes included in the study (notably *MMP20*, *ENAM* and *AMBN*), meaning that the sensitivity of variant detection in these genes will have been reduced. Also, ExomeDepth, a tool primarily designed for WES data, may not be optimal for smMIP data, meaning larger structural variants or complex rearrangements may have been missed. Additional probes targeting low coverage regions and new genes and variants implicated in AI in the literature could be added to the existing reagent, while new pathogenicity prediction tools could be added to the analysis pipeline, making smMIPs a flexible diagnostics tool for AI research. Nevertheless, WES or WGS in cases not solved by smMIPs is likely to reveal further variants that were refractory to detection due to the capture, sequencing or analysis pipeline used in this study, as well as by identifying intragenic and intronic variants which are not covered by the smMIP reagent.

By assessing the findings of this study in the context of another recently published AI cohort [49], we note that both this and the previous study identify dominant AI as accounting for nearly half of isolated AI cases, with recessive disease at approximately 40% and the remainder being solved as X-linked disease. However, screening results may reflect biases in sampling, current knowledge or differences in the populations screened. Previous cohort studies have suggested that dominant AI is much more common than recessive disease [48, 50]. The relatively high frequency of recessive AI in the cohort studied here may reflect the inclusion of families from the Yorkshire Pakistani community, which has a high level of first cousin marriage and consequent increased risk of recessive disease [51]. The spectrum of variants and frequencies of the different forms of AI revealed by this study and that of Bloch-Zupan et al. [49] are broadly similar, but include some notable differences. This study found dominant *COL17A1* variants to be the most common cause of isolated AI, accounting for 17% of cases, whereas the other study found recessive *MMP20* variants to be the leading cause. Data on *COL17A1*-related AI from this study is included in a more detailed study reported elsewhere [52]. Furthermore, our findings reveal that variants in *AMBN* account for 8% of solved cases, compared to only 3% in the other study, and cause both recessive and dominant isolated AI. These findings are also described in more detail elsewhere [53].

The premature termination codon variant *FAM83H*: c.601C>T, p.(Gln201^∗^), with a CADD score of 40, was present as a heterozygous variant in three families within this cohort. However, this variant (SNP identifier rs189033490) is classified as benign by ACMG criteria (BS1 and BP6). The frequency in gnomAD (0.0024) is considered too high for this variant to be a plausible cause of dominantly inherited AI, and the ClinVar entry (Accession: VCV000402847.9) reports conflicting classifications of pathogenicity. Premature termination codons are the most commonly observed pathogenic variants in *FAM83H*, but only those in the large last exon (Exon 5) are thought to cause AI [54], while this variant is in the second coding exon (Exon 3). AI patients with this variant were therefore not considered solved.

Another notable finding of this study is the identification of potentially disease-causing variants in both *COL17A1* and *MMP20* in five individuals from four families. Multiple, potentially pathogenic variants can be expected to be an increasingly common finding as more genetic data becomes available. There is a need to better understand this situation in what is considered to be a monogenetic condition. It is plausible that, in some instances, genetic variants are essential in more than one gene for AI to occur, most likely as a digenic model. An alternative explanation is that variant(s) in one gene are sufficient to cause AI, but that phenotype can be modified by variants in one or more other genes. This may explain the variation sometimes seen between individuals with the same genotype. These possibilities require further active exploration in a large patient cohort within the context of clinic-pathological correlation, including consideration of the mode of inheritance and the potential contributions to phenotype or severity from systemic illness or environmental causes.

Digenic AI has been suggested in three previous studies. One showed the cosegregation of *ENAM* and *LAMA3* variants with AI through six meioses in a family [55]. The second reported a single case with *COL17A1* and *LAMA3* variants [56]. The third consisted of a father with AI thought to be due to a *LAMA3* VOUS and a son with more severe AI, who carried the same *LAMA3* variant but was also a compound heterozygote for two likely pathogenic *MMP20* variants [57]. There is also published evidence that specific heterozygous variants in each of *Mmp20* and *Klk4* caused an enamel phenotype in mice, but a single heterozygous variant in either gene did not [58].

Though speculative, these reports suggest that some AI might in fact be polygenic rather than the typical single gene Mendelian model. With four probands carrying allele combinations involving both *MMP20* and *COL17A1*, this study could be interpreted as providing further circumstantial evidence for such an effect. However, there is currently no evidence of a direct functional link between these two proteins. Furthermore, AI in each case is fully explained by one genotype (the *COL17A1* genotype in Families 4 and 18 and the *MMP20* genotype in Families 25 and 62, as shown in Figure 2) without the need to invoke any contribution from the other gene, and the phenotypes observed in these families are consistent with previously documented phenotypes for variants in these genes. Accordingly, in these instances, any modification impact by the second gene is likely to have been minimal.

## 5. Conclusions

In summary, we have developed and validated a flexible smMIP reagent for rapid, high-throughput, cost-effective screening for variants in 19 genes known to be implicated in isolated AI. Intended as a prescreen for AI cases, its use in a cohort of individuals with AI resulted in molecular diagnoses for 63 probands and their families. This analysis confirmed dominant inheritance as the most common mode of inheritance in AI, with *COL17A1* and *FAM83H* variants as the most common underlying causes in this cohort. The success of this approach demonstrated here highlights the power of smMIPs, gives insights into the epidemiology of isolated, nonsyndromic AI and provides a reagent that is now available to AI research groups around the world. Importantly, the composition of the reagent can be adapted to add further genes implicated in both isolated and syndromic forms of AI, thereby accounting for future developments in the field.

## Figures and Tables

**Table 1:** Genes included in the smMIP reagent.

**HGNC gene name**	**HGNC gene symbol**	**Transcript**	**OMIM identifier**	**Genomic coordinates**	**Cytoband**
Laminin Subunit Beta 3	*LAMB3*	NM_000228.3	150310	chr1:209,788,215-209,825,770	1q32.2
Integrin Subunit Beta 6	*ITGB6*	NM_000888.5	147558	chr2:160,956,182-161,056,783	2q24.2
Amelotin	*AMTN*	NM_001286731.2	610912	chr4:71,384,286-71,398,460	4q13.3
Ameloblastin	*AMBN*	NM_016519.6	601259	chr4:71,457,973-71,473,005	4q13.3
Enamelin	*ENAM*	NM_031889.3	606585	chr4:71,494,461-71,512,541	4q13.3
Odontogenesis-associated phosphoprotein	*ODAPH*	NM_001206981.2	614829	chr4:76,481,276-76,491,095	4q21.1
Family with sequence similarity 83 member H	*FAM83H*	NM_198488.5	611927	chr8:144,806,103-144,815,949	8q24.3
Collagen Type XVII Alpha 1 chain	*COL17A1*	NM_000494.4	113811	chr10:105,791,044-105,845,638	10q25.1
RELT TNF receptor	*RELT*	NM_152222.2	611211	chr11:73,087,444-73,108,519	11q13.4
Matrix metallopeptidase 20	*MMP20*	NM_004771.4	604629	chr11:102,447,563-102,496,063	11q22.2
G protein-coupled receptor 68	*GPR68*	NM_001177676.2	601404	chr14:91,698,876-91,711,048	14q32.11
Solute carrier Family 24 Member 4	*SLC24A4*	NM_153646.4	609840	chr14:92,789,509-92,967,825	14q32.12
WD repeat domain 72	*WDR72*	NM_182758.4	613214	chr15:53,805,938-54,051,860	15q21.3
Sp6 transcription factor	*SP6*	NM_199262.3	608613	chr17:45,922,274-45,933,063	17q21.32
Distal-less homeobox 3	*DLX3*	NM_005220.3	600525	chr17:48,067,369-48,072,588	17q21.33
FAM20A golgi associated secretory pathway pseudokinase	*FAM20A*	NM_017565.4	611062	chr17:66,531,257-66,597,508	17q24.2
Acid phosphatase 4	*ACP4*	NM_033068.3	606362	chr19:51,293,672-51,298,476	19q13.33
Kallikrein-related peptidase 4	*KLK4*	NM_004917.5	603767	chr19:51,409,607-51,414,651	19q13.41
Amelogenin X-linked	*AMELX*	NM_182680.1	300391	chrX:11,311,533-11,318,881	Xp22.2

*Note:* Genomic coordinates are provided with respect to the specified transcripts and are according to the human reference genome build hg19.

Abbreviations: HGNC, HUGO Gene Nomenclature Committee; OMIM, Online Mendelian Inheritance in Man.

**Table 2:** Details of the variants identified in the X-linked families.

**Family ID**	**HGNC gene symbol**	**Genomic nomenclature**	**Transcript nomenclature**	**Protein nomenclature**	**Zygosity**	**CADD score**	**gnomAD frequency**	**PolyPhen-2/SIFT/SpliceAI**	**Variant classification**	**Reference**
8	*AMELX*	chrX:g.11314944G>A	c.100G>A	p.(Glu34Lys)	Heterozygous	33	Absent	0.999/0.004	VOUS	Novel variant
9	*AMELX*	chrX:g.11316220T>C	c.103-3T>C	p.?	Hemizygous	15	Absent	0.35 gain	LP	[28]
21	*AMELX*	chrX:g.11316954del	c.473del	p.(Pro158Hisfs∗31)	Hemizygous		Absent	N/A	P	[29]
27	*AMELX*	chrX:g.11316366del	c.155del	p.(Pro52Leufs∗2)	Heterozygous		Absent	N/A	P	[30]
32	*AMELX*	chrX:g.11316220T>C	c.103-3T>C	p.?	Heterozygous	15	Absent	0.35 gain	LP	[28]
38	*AMELX*	chrX:g.11316366del	c.155del	p.(Pro52Leufs∗2)	Hemizygous		Absent	N/A	P	[30]
65	*AMELX*	chrX:g.11316927A>C	c.446A>C	p.(Gln149Pro)	Hemizygous	19	0.00001	0.007/0.182	VOUS	Novel variant

*Note:* The PolyPhen-2 score ranges from 0.0 (*tolerated*) to 1.0 (*deleterious*). A SIFT score ranges from 0 (typically below 0.05 is considered *deleterious*) to 1 (*tolerated*). The SpliceAI score ranges from 0 to 1, with higher scores indicating a greater likelihood that the variant affects splicing. PolyPhen-2/SIFT scores are included for exonic missense variants, while SpliceAI scores are provided for intronic and exonic variants predicted to alter splicing. Nomenclature is reported according to the human reference genome build hg19.

Abbreviations: CADD, combined annotation-dependent depletion (v.1.3); gnomAD, genome aggregation database (v.2.1.1); HGNC, HUGO Gene Nomenclature Committee; LP, likely pathogenic; novel variant, variant identified in this study; P, pathogenic; reference, previous reporting of the variant; VOUS, variant of uncertain significance.

**Table 3:** Details of the variants identified in the dominant families.

**Family ID**	**HGNC gene symbol**	**Genomic nomenclature**	**Transcript nomenclature**	**Protein nomenclature**	**CADD score**	**gnomAD frequency**	**PolyPhen-2/SIFT/SpliceAI**	**Variant classification**	**Reference**
1	*COL17A1*	chr10:g.105811247C>T	c.2030G>A	p.(Gly677Asp)	26	Absent	0.998/0.001	LP	Novel variant
4	*COL17A1*	chr10:g.105798865del	c.2912del	p.(Pro971Glnfs∗95)	33	Absent	N/A	LP	Novel variant
5	*DLX3*	chr17:g.48069185_48069186del	c.561_562del	p.(Tyr188Glnfs∗13)		Absent	N/A	P	[31]
7	*COL17A1*	chr10:g.105796271G>A	c.3397C>T	p.(Arg1133Cys)	33	Absent	0.661/0.092	VOUS	Novel variant
11	*COL17A1*	chr10:g.105796802C>T	c.3277+1G>A	p.?	35	Absent	0.80 loss	P	Novel variant
13	*COL17A1*	chr10:g.105811266C>T	c.2011G>A	p.(Gly671Ser)	25	Absent	0.762/0.001	LP	Novel variant
14	*ENAM*	chr4:g.71503505A>T	c.535-2A>T	p.?	33	Absent	0.90 Loss	LP	[32]
16	*AMBN*	chr4:g.71465278C>G	c.209C>G	p.(Ser70∗)	36	0.00010	N/A	P	Novel variant
18	*COL17A1*	chr10:g.105830245_105830254del	c.541_550del	p.(Asn181Profs∗13)	28	Absent	N/A	P	Novel variant
22	*FAM83H*	chr8:g.144810257G>T	c.1374C>A	p.(Tyr458∗)	38	Absent	N/A	P	[33]
24	*COL17A1*	chr10:g.105795287del	c.3456del	p.(Pro1154Leufs∗97)	21	0.00002	N/A	P	Novel variant
26	*COL17A1*	chr10:g.105793715_105793716del	c.4147_4148del	p.(Ser1383Hisfs∗71)	34	Absent	N/A	P	Novel variant
30	*ENAM*	chr4:g.71497387del	c.55del	p.(Val19Tyrfs∗6)	33	Absent	N/A	LP	Novel variant
33	*ENAM*	chr4:g.71497439del	c.107del	p.(Asn36Ilefs∗22)		Absent	N/A	P	[34]
34	*ENAM*	chr4:g.71501548G>A	c.472-1G>A	p.?	25	Absent	0.87 loss/gain	LP	Novel variant
35	*ENAM*	chr4:g.71497439del	c.107del	p.(Asn36Ilefs∗22)		Absent	N/A	P	[34]
36	*COL17A1*	chr10:g.105816859C>A	c.1339G>T	p.(Gly447Cys)	24	0.00070	1/0.001	VOUS	Novel variant
39	*FAM83H*	chr8:g.144809494G>A	c.2137C>T	p.(Gln713∗)	36	Absent	N/A	LP	Novel variant
40	*AMBN*	chr4:g.71465278C>G	c.209C>G	p.(Ser70∗)	36	0.00010	N/A	P	Novel variant
41	*DLX3*	chr17:g.48072078G>C	c.285C>G	p.(Tyr95∗)	36	Absent	N/A	P	Novel variant
43	*FAM83H*	chr8:g.144810439G>A	c.1192C>T	p.(Gln398∗)	36	Absent	N/A	LP	[35]
44	*AMBN*	chr4:g.71459104G>A	c.76G>A	p.(Ala26Thr)	26	Absent	1/0.000	LP	Novel variant
45	*FAM83H*	chr8:g.144810277G>A	c.1354C>T	p.(Gln452∗)	37	Absent	N/A	LP	[36]
48	*COL17A1*	chr10:g.105831793G>A	c.460C>T	p.(Arg154∗)	36	Absent	N/A	P	[37]
49	*COL17A1*	chr10:g105795287del	c.3456del	p.(Pro1154Leufs∗97)	21	0.00002	N/A	P	Novel variant
55	*FAM83H*	chr8:g.144810268G>A	c.1363C>T	p.(Gln455∗)	37	Absent	N/A	P	[38]
56	*COL17A1*	chr10:g.105795035G>A	c.3605C>T	p.(Ser1202Leu)	27	0.00001	0.061/0.009	VOUS	Novel variant
57	*FAM83H*	chr8:g.144810658G>A	c.973C>T	p.(Arg325∗)	36	Absent	N/A	P	[39]
63	*FAM83H*	chr8:g.144810710_144810711del	c.923_924del	p.(Leu308Argfs∗16)		Absent	N/A	LP	[40]

*Note:* All variants were identified in a heterozygous state. The PolyPhen-2 score ranges from 0.0 (*tolerated*) to 1.0 (*deleterious*). A SIFT score ranges from 0 (typically below 0.05 is considered *deleterious*) to 1 (*tolerated*). The SpliceAI score ranges from 0 to 1, with higher scores indicating a greater likelihood that the variant affects splicing. PolyPhen-2/SIFT scores are included for exonic missense variants, while SpliceAI scores are provided for intronic and exonic variants predicted to alter splicing. Nomenclature is reported according to the human reference genome build hg19.

Abbreviations: CADD, combined annotation-dependent depletion (v.1.3); gnomAD, genome aggregation database (v.2.1.1); HGNC, HUGO Gene Nomenclature Committee; LP, likely pathogenic; novel variant, variant identified in this study; P, pathogenic; reference, previous reporting of the variant; VOUS, variant of uncertain significance.

**Table 4:** Details of the variants identified in the recessive families.

**Family ID**	**HGNC gene symbol**	**Genomic nomenclature**	**Transcript nomenclature**	**Predicted protein nomenclature**	**CADD score**	**gnomAD frequency**	**PolyPhen-2/SIFT/SpliceAI**	**Variant classification**	**Reference**
2	*ACP4*	chr19:g.51294940C>T	c.331C>T	p.(Arg111Cys)	26	0.00012	1/0.000	LP	[41]
3	*FAM20A*	chr17:g.66538249_66538252del	c.987_990del	p.(Cys330Alafs∗51)	33	Absent	N/A	LP	Novel variant
6	*MMP20*	chr11:g.102480660C>G	c.625G>C	p.(Glu209Gln)	30	0.00006	1/0.002	LP	[42]
12	WDR72	chr15:g.53908077dup	c.2332dup	p.(Met778Asnfs∗4)	24	0.00001	N/A	P	[43]
		chr15:g.54025229G>A	c.118C>T	p.(Gln40∗)	40	0.00002	N/A	P	Novel variant
15	*MMP20*	chr11:g.102465490T>A	c.954-2A>T	p.?	25	0.00110	0.84 loss	P	[44]
17	*MMP20*	chr11:g.102480660C>G	c.625G>C	p.(Glu209Gln)	27	0.00007	0.999/0.002	P	[42]
19	*AMBN*	chr4:g.71465278C>G	c.209C>G	p.(Ser70∗)	36	0.00010	N/A	P	Novel variant
20	*MMP20*	chr11:g.102480660C>G	c.625G>C	p.(Glu209Gln)	27	0.00007	0.999/0.002	P	[42]
23	*SLC24A4*	Deletion of Exons 15–17					N/A		[45]
25	*MMP20*	chr11:g.102479824T>A	c.655A>T	p.(Asn219Tyr)	27	Absent	1/0.000	VOUS	Novel variant
28	*AMBN*	chr4:g.71465278C>G	c.209C>G	p.(Ser70∗)	36	0.00010	N/A	P	Novel variant
		chr4:g.71467135T>C	c.295T>C	p.(Tyr99His)	26	0.00008	1/0.000	LP	Novel variant
37	*ITGB6*	chr:2:g.161052847C>A	c.226G>T	p.(Glu76∗)	39	0.00001	N/A	P	Novel variant
42	*RELT*	chr:11g.73101947T>C	c.268T>C	p.(Cys90Arg)	26	Absent	0.995/0.000	LP	Novel variant
46	*MMP20*	chr11:g.102479803G>A	c.676C>T	p.(His226Tyr)	28	Absent	1/0.000	LP	Novel variant
47	*MMP20*	chr11:g.102465490T>A	c.954-2A>T	p.?	25	0.00110	0.84 loss	P	Novel variant
50	*ACP4*	chr19:g.51294940C>T	c.331C>T	p.(Arg111Cys)	26	0.00012	1/0.000	P	[41]
		chr19:g.51295044del	c.435del	p.(Val146Trpfs∗7)	32	Absent	N/A	P	Novel variant
51	*SLC24A4*	chr14:g.92920382T>C	c.1019T>C	p.(Leu340Pro)	25	Absent	1/0.950	VOUS	Novel variant
52	*WDR72*	chr15:g.53907717G>A	c.2686C>T	p.(Arg896∗)	36	0.00003	N/A	P	Novel variant
53	*FAM20A*	chr17:g.66535488G>A	c.1351C>T	p.(Gln451∗)	47	Absent	N/A	LP	Novel variant
54	*WDR72*	chr15:g.53994432_53994433del	c.1467_1468del	p.(Val491Aspfs∗8)		0.00007	N/A	P	[46]
58	*MMP20*	chr11:g.102465490T>A	c.954-2A>T	p.?	25	0.00110	0.84 loss	P	[44]
		chr11:g.102477286del	c.933del	p.(Glu311Aspfs∗59)	32	0.00001	N/A	P	Novel variant
59	*WDR72*	chr15:g.53992117_53992118del	c.1600_1601del	p.(Cys534Argfs∗2)		Absent	N/A	P	Novel variant
		chr15:g.53907897C>A	c.2506G>T	p.(Glu836∗)	37	Absent	N/A	P	Novel variant
60	*ACP4*	chr19:g.51297211T>C	c.845T>C	p.(Met282Thr)	27	Absent	0.985/0.001	VOUS	Novel variant
61	*FAM20A*	chr17:g.66551883G>A	c.406C>T	p.(Arg136∗)	39	0.00004	N/A	P	[47]
		chr17:g.66538120A>C	c.1109+6T>G	p.?	23	Absent	0.67 loss	LP	Novel variant
62	*MMP20*	chr11:g.102465490T>A	c.954-2A>T	p.?	25	0.00110	0.84 loss	P	[44]
64	*ENAM*	chr4:g.71508226G>A	c.1083G>A	p.(Trp361∗)	36	Absent	N/A	LP	Novel variant
66	*WDR72*	chr15:g.54003125C>T	c.883G>A	p.(Ala295Thr)	20	Absent	0.593/0.004	LP	Novel variant

*Note:* Families reported with a single variant are homozygous, while families reported with two variants are compound heterozygous. The PolyPhen-2 score ranges from 0.0 (*tolerated*) to 1.0 (*deleterious*). A SIFT score ranges from 0 (typically below 0.05 is considered *deleterious*) to 1 (*tolerated*). The SpliceAI score ranges from 0 to 1, with higher scores indicating a greater likelihood that the variant affects splicing. PolyPhen-2/SIFT scores are included for exonic missense variants, while SpliceAI scores are provided for intronic and exonic variants predicted to alter splicing. Nomenclature is reported according to human reference genome build hg19.

Abbreviations: CADD, combined annotation-dependent depletion (v.1.3); gnomAD, genome aggregation database (v.2.1.1); HGNC, HUGO Gene Nomenclature Committee; LP, likely pathogenic; novel variant, variant identified in this study; P, pathogenic; reference, previous reporting of the variant; VOUS, variant of uncertain significance.

**Table 5:** Details of the variants identified in the four participants presented with potentially relevant variants in two genes known to be associated with isolated AI. None of the variants have previously been reported by others. Families 4 and 18 have been reported by us as solved based on the presence of *COL17A1* variants correlated to the clinical features and patterns of inheritance in the families [52]. Families 25 and 62 are considered solved, reflecting the presence of *MMP20* variants correlated to clinical features and patterns of inheritance in the families.

**Family ID**	**HGNC gene symbol**	**Genomic nomenclature**	**Transcript nomenclature**	**Predicted protein nomenclature**	**Zygosity**	**CADD score**	**gnomAD frequency**	**Pathogenicity**	**ClinVar ID**
25	*COL17A1*	chr10:g.105799724A>T	c.2788+7T>A	p.?	Het	17	0.00006	VOUS	
	*MMP20*	chr11:g.102479824T>A	c.655A>T	p.(Asn219Tyr)	Hom	26	Absent	VOUS	
62	*COL17A1*	chr10:g.105830262G>A	c.529C>T	p.(Arg177Trp)	Het	27	0.00005	VOUS	
	*MMP20*	chr11:g.102465490T>A	c.954-2A>T	p.?	Hom	25	0.00110	P	
4	*COL17A1*	chr10:g.105798865del	c.2912del	p.(Pro971Glnfs∗95)	Het		Absent	LP	VCV002572040.2
	*MMP20*	chr11:g.102495959G>A	c.92C>T	p.(Pro31Leu)	Het	21	0.00460	LB	VCV000301957.9
		chr11:g.102477309C>T	c.910G>A	p.(Ala304Thr)	Het	25	0.00156	VOUS	VCV000301942.9
18	*COL17A1*	chr10:g.105830245_105830254del	c.541_550del	p.(Asn181Profs∗13)	Het	28	Absent	P	VCV002572036.2
	*MMP20*	chr11:g.102495959G>A	c.92C>T	p.(Pro31Leu)	Het	21	0.00460	LB	VCV000301957.9
		chr11:g.102477309C>T	c.910G>A	p.(Ala304Thr)	Het	25	0.00156	VOUS	VCV000301942.9

*Note:* Genomic coordinates are provided according to the human reference genome build hg19.

Abbreviations: CADD, combined annotation-dependent depletion (v.1.3); ClinVar, clinical interpretation of DNA sequence variants; gnomAD, genome aggregation database (v.2.1.1); Het, heterozygous; HGNC, HUGO Gene Nomenclature Committee; Hom, homozygous; LB, likely benign; LP, likely pathogenic; P, pathogenic; VOUS, variant of uncertain significance.

## Data Availability

The data that support the findings of this study are available on request from the corresponding author. The data are not publicly available due to privacy or ethical restrictions.
